# Nutritional programming in Nile tilapia *(Oreochromis niloticus)*: Effect of low dietary protein on growth and the intestinal microbiome and transcriptome

**DOI:** 10.1371/journal.pone.0292431

**Published:** 2023-10-04

**Authors:** Courtney A. Deck, Scott A. Salger, Hannah M. Reynolds, Michael D. Tada, Madeline E. Severance, Peter Ferket, Hillary S. Egna, Mst. Kaniz Fatema, Shahroz M. Haque, Russell J. Borski

**Affiliations:** 1 Department of Biological Sciences, North Carolina State University, Raleigh, NC, United States of America; 2 School of Sciences, Barton College, Wilson, NC, United States of America; 3 Department of Poultry Science, North Carolina State University, Raleigh, NC, United States of America; 4 Department of Fisheries and Wildlife, Oregon State University, Corvallis, OR, United States of America; 5 Faculty of Fisheries, Bangladesh Agricultural University, Mymensingh, Bangladesh; CIFRI: Central Inland Fisheries Research Institute, INDIA

## Abstract

Nutritional programming is the idea that early nutrient contributions can influence organismal structure or function and is documented in a variety of vertebrates, yet studies in fish are largely lacking. Tilapia are an important foodfish, with global production having increased rapidly since the 1990s. They exhibit high disease-resistance and grow well on formulated feeds which makes them an ideal aquaculture species, however incorporating high quality proteins into feeds can be costly. As feed constitutes 50–70% of total production costs in aquaculture, reducing protein content could curb these costs and increase revenue. Thus, we examined the effects of feeding Nile tilapia (*O*. *niloticus*) fry a restricted protein diet for the first 7–21 days on growth, gut microbial flora, and the intestinal transcriptome. Fish were fed either a 25% restricted or 48% control crude protein starter (ST) diet for up to 21 days and then switched to a 25% or 38% control crude protein growout (GO) diet. Fish fed a 25% ST diet for 14 days followed by a 38% GO diet had significantly higher lengths and weights and better feed efficiency than fish fed the control 48% ST and 38% GO diet after 56 days of culture. Growth of fry on the 25% ST, 7-day/38% GO and the 25% ST,7-day/25% GO diets did not differ from the those fed the control protein diets, while fish fed the 25% ST diet for 21 days had significantly lower growth and survival rates. We observed no significant differences in either alpha or beta diversity of the gut microbial flora between diets, however species richness (Shannon Index) was higher in fry fed the 25% protein ST diet regardless of the GO diet. Similarly, fish fed the 25% ST diet for 14 days followed by the 38% GO diet had minimal changes to the intestinal transcriptome relative to fish fed the control 48% ST and 38% GO diet. However, those fed 25% ST and GO diets for the entire 56 days exhibited substantial differences in the gut transcriptome from other groups showing gene expression profiles characteristic of detrimental changes to gut physiology, protein metabolism and immune function. Results suggest protein restriction for up to 14 days early in development leads to enhanced growth and feed efficiency with minimal effects on gut microbes or intestinal function. Protein restriction beyond this period appears detrimental to fish growth and health as underscored by expression of disease related genes and higher mortality rates.

## Introduction

Nutritional programming (also known as nutritional conditioning or imprinting) is the concept that dietary nutrient contributions or other environmental factors experienced early in development influence organismal structure or function and can thus lead to life-long changes in key elements of an animal’s physiology, including growth, metabolism, cardiovascular function, and life span [1]. Early nutrition can alter the function of the gastrointestinal tract by affecting the development of the mucosa to protect against pathogens and the ability to take up nutrients [2]. Thus, altering nutrient levels early in development can have major repercussions for immune function, the absorption of nutrients, and the efficiency with which these nutrients are used by the body. Further, nutritional programming can alter the composition of the intestinal microbiota which has emerged as a key factor in health and the prevention of diseases due to the ability of these microorganisms to synthesize vitamins, break down toxins, and act as antimicrobial agents against pathogenic bacteria [3, 4]. Nutritional programming has been of great interest in human health with regards to the effect of pre-natal maternal nutrition as well as post-natal diet composition on offspring development and the pathogenesis of diseases such as obesity and diabetes later in life [1, 3]. However, research to date indicates that this phenomenon is likely widespread across vertebrate taxa and thus it could have substantial implications in agriculture and aquaculture for enhancing the growth and health of food animals and also reducing environmental impacts [5].

Studies suggest that consuming low levels of a particular nutrient or undergoing periods of fasting can lead to compensatory mechanisms that enhance nutrient uptake efficiency by the intestine at the next feeding period [6–9]. Broiler chickens fed a phosphorous- and calcium-deficient diet for the first 18 days post-hatch had an increased ability to absorb both minerals from their diets at 32 days post-hatch [10]. They also exhibited compensatory growth and compensatory improvements to bone density and composition following the 18-day deprivation period [10]. Similarly, chickens fed a low phosphorous diet for the first 90 hours post-hatch had higher intestinal mRNA levels of the sodium-phosphorous cotransporter (NaPcoT) at 38 days, suggesting a compensatory mechanism to maximize uptake of this nutrient [7]. Other studies show that increasing the dietary proportion of n-3 fatty acids in either the breeder hens or the chicks immediately post-hatch reduces the production of pro-inflammatory compounds during the growout phase, which may allow more energy to be allocated toward growth by preventing inflammation-related disorders [11, 12; reviewed by 13].

A few studies have also investigated the effect of early nutrient adjustments in fish. European sea bass juveniles (*Dicentrarchus labrax*) that had initially been provided with a diet low in highly unsaturated fatty acids (HUFA) metabolized lipids more efficiently than those fed a high HUFA diet [14], while rainbow trout (*Oncorhynchus mykiss*) juveniles fed a hyperglucidic diet for the first 5 days of feeding had higher mRNA levels of enzymes involved in carbohydrate digestion at 110 days post-hatch [15]. The latter group also showed that a high glucose, low protein diet caused long-term increases in the expression of glycolytic enzymes and decreases in enzymes involved in amino acid catabolism in the muscle relative to those that received a high protein, glucose-free diet at first-feeding [16]. Further, the high carbohydrate diet did not affect growth during the 105-day growout period, but it did alter the intestinal fungal community, indicating that microbial programming is possible in fish as it is in mammals [16]. Izquierdo et al. [17] also showed that programming is possible through alterations to broodstock diets. Gilthead sea bream (*Sparus aurata*) progeny from broodstock fed diets high in vegetable oils had higher growth and feed utilization when fed a similar diet as juveniles than to those from broodstock that had been fed a diet high in fish oils [17]. A recent study suggests that exposure to a high carbohydrate diet following first feeding can improve subsequent growth of juvenile Nile tilapia (*Oreochromis niloticus*) [18].

The effectiveness of applying nutritional programming to tilapia culture remains poorly understood, this despite the high rate of increase in the global production of farmed Nile tilapia [19, 20]. Further, the mechanisms underlying how nutritional programming can potentially alter intestinal microflora or achieve equivalent production yields despite reductions in essential nutrients is unknown. Tilapia are an ideal aquaculture species as they are resistant to disease and water quality issues relative to many other farmed species, grow well on formulated feeds, and can tolerate lower protein levels relative to carnivorous species [21]. With feed constituting 50–70% of total variable production costs for farmed fish and protein being the costliest component of commercial feeds [21], the ability to utilize feeds with a lower protein content could greatly reduce costs and enhance production efficiency. There is some evidence that during periods of fasting, nutrient uptake efficiency in the intestine is intrinsically enhanced [6, 8]. Thus, decreasing the amount of select nutrients early in life may increase the uptake and utilization of those nutrients during the growout phase of fish culture.

Here, we investigated whether nutritional programming can be applied to Nile tilapia (*Oreochromis niloticus*) by restricting dietary protein levels early in development. We evaluated the effects of the restricted diet on growth and survival and used a transcriptomic approach to determine the suite of genes in the intestine that are affected by early protein restriction. Additionally, we performed a marker gene microbiome survey to evaluate the effects of nutritional programming on microbial colonization of the gut. The establishment of beneficial microflora can affect nutrient availability and gut health [22] and the emerging field of metagenomics has substantial implications for sustainable aquaculture as diet, feeding strategy, and other environmental factors strongly influence the diversity and constitutive abundance of intestinal microbiota in both humans and fish [23–26].

## Materials and methods

### Animals

Post-yolk sac Nile tilapia fry (*Oreochromis niloticus*) were obtained from the Louisiana Specialty Aquafarm (Harvey, LA, USA) and transferred to the Grinnell’s Fish Laboratory at North Carolina State University (NCSU; Raleigh, NC, USA) where they were stocked in 10 L AHAB tanks (Aquatic Habitats, Pentair Aquatic Ecosystems; Cary, NC, USA) at a density of 38 fry/L (0.46 g/L) at 26°C with a 12h light:12h dark photoperiod. All experiments and sampling were performed in accordance with the NCSU Institutional Animal Care and Use Committee (17-003-0). All sampled animals were euthanized using buffered tricaine methanesulfonate (MS-222, Pentair Aquatic Ecosystems) following standard procedures.

### Nutritional programming

Restricted (25% starter and 25% growout) and control (48% starter and 38% growout) crude protein (CP) diets were formulated by Integral Fish Foods (Albany, IN, USA) using nutritional profiles outlined by Mjoun et al. [27] as guidelines and mixed at Carolina Classic Catfish (Winterville, NC, USA). The control CP diet reflects that frequently used for tilapia production in recirculating aquaculture systems. The final nutrient compositions for each diet are shown in S1 Table. The feeds were then extruded by the Bozeman Fish Technology Centre (Bozeman, MT, USA). The extruded feed was processed at 1.2 mm in diameter for the starter diets and 2 mm for the growout diets. The starter diets were crumbled in the lab to produce smaller pellets for initial feeding of fry. Soybean and menhaden fish oils were added to the extruded feeds prior to feeding to limit spoilage.

**Fig 1:**
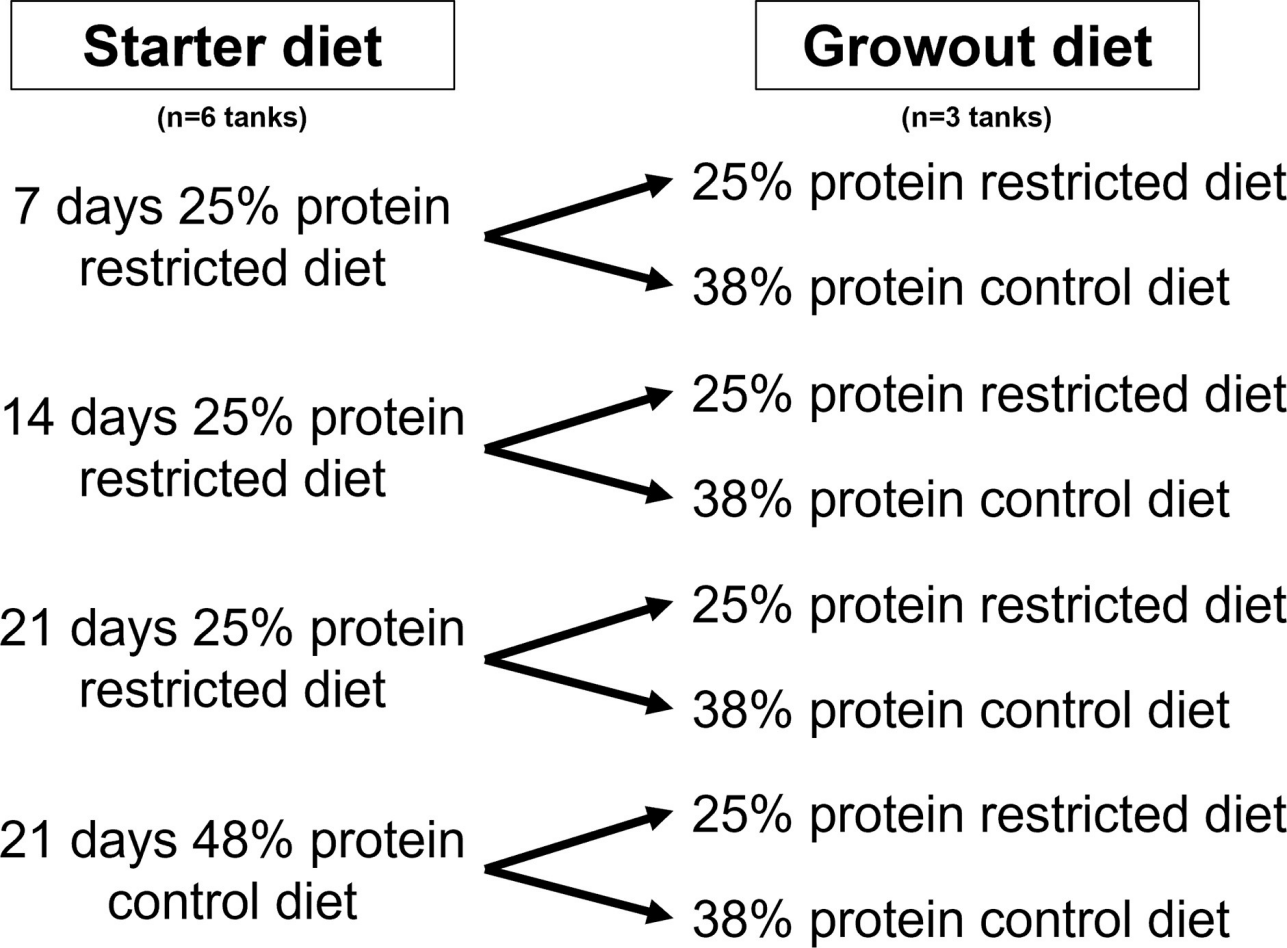
Experimental design for nutritional programming with different dietary protein levels.

Fry were fed either a control (48% CP) for 21 days or a restricted (25% CP) protein starter (ST) diet for 7, 14, or 21 days. They were then switched to a control (38% CP) or restricted (25% CP) protein growout (GO) diet for the remainder of the 56-day study period (Fig 1). Each treatment was replicated in 3 separate tanks. Fish were fed 3 times daily to satiation for the first 28 days and then 2 times daily to satiation from 29–56 days. The feed was weighed prior to and following each feeding to quantify the amount offered. The tanks were cleaned daily to prevent the fish from consuming algae or feces. Fry were sub-sampled on a weekly basis for length and weight measurements (n = 10 per tank, 30 total per treatment). Feed conversion was estimated based on the ratio of the amount of feed offered to the weight gained by the fry after 7, 14, 21, 28, and 56 days of the trial.

### Microbiome analysis

Fry were sub-sampled at 7, 14, 21, 28, 35, and 56 days after the start of the study. Fish <15 mm in length (prior to 21 days of the experiment) were too small for intestinal dissection so whole fry were rinsed in 70% ethanol and sterile deionized water to eliminate external microbes on the skin of the fish [28, 29] before being placed in BashingBead™ buffer from a Quick-DNA Fecal/Soil Microbe Kit (Zymo Research, Irvine, CA, USA). For fish >15 mm in length (21 days and beyond), fecal matter was collected from the posterior intestine (colon) and placed into buffer. All samples were then bullet homogenized and genomic DNA (gDNA) extracted according to the manufacturer’s instructions (Zymo Research, Irvine, CA, USA). Samples were pooled according to treatment group to offset potential variability of microbiota within individuals and allow us to focus on common patterns among the population as a whole (3 pooled samples per tank from triplicate tanks, each pool consisting of 2 fish, n = 9 pooled samples or 18 individual fish per treatment). The gDNA concentration and quality were determined by Nanodrop (Thermo Fisher Scientific, Waltham, MA, USA) and the extracted gDNA was stored at -20°C for sequencing library preparations.

Prokaryotic 16S rRNA gene amplicons were prepared following the 16S Metagenomic Sequencing Library Preparation protocol for the Illumina MiSeq system with some modifications (Illumina, Inc., San Diego, CA, USA). Primers were designed to amplify the V3 to V5 regions of prokaryotic 16S rRNA [30–32] with overhang adapter sequences compatible with the Illumina index and sequencing adapters (S2 Table). This allowed for double indexing to increase the accuracy of the multiplexed reads. Amplicon PCR was used to amplify the region of interest from the gDNA extracted from the tilapia fecal material samples. The PCR consisted of 1 cycle of 95°C for 3 min; 25 cycles each of 95°C for 30 s, 55°C for 30 s, and 72°C for 30 s; 1 cycle of 72°C for 5 min; and then held at 4°C. Clean-up of the PCR amplicon products to remove free primers and primer dimers was performed using Agencourt AMPure XP beads (Beckman Coulter, Inc., Atlanta, GA, USA) and freshly prepared 80% ethanol for clean-up. Following amplicon clean-up, PCR was performed to attach indices to the amplicon PCR products. Dual-index primers were designed so that samples could be multiplexed in one MiSeq lane (S2 Table). Index PCR was performed as follows: 1 cycle of 95°C for 3 min; 8 cycles each of 95°C for 30 s, 55°C for 30 s, and 72°C for 30 s; 1 cycle of 72°C for 5 min; and hold at 4°C. Clean-up of the PCR index products was performed as above. All indexed amplicon concentrations were normalized and amplicons pooled into a single tube. The pooled library was checked for quality and quantified using an Agilent ScreenTape on the Agilent 2200 Tapestation (Agilent Technologies, Inc., Santa Clara, CA, USA). The library was diluted and combined with a PhiX Control library (v3) (Illumina) at 10%. The library was sequenced on an Illumina v3 300PE MiSeq run at the NCSU Genomic Sciences Laboratory (Raleigh, NC, USA), using standard sequencing protocols. Base calls were generated on-instrument during the sequencing run using the MiSeq Real Time Analysis (RTA 1.18.54) software and Fastq generation; demultiplexing, adapter trimming, and quality filtering were performed by the MiSeq Reporter Software (2.4 and 2.5.1). The library was run on two lanes to increase the number of reads for each sample (9 samples/treatment).

The resulting demultiplexed reads were processed using the QIIME 2 microbiome analysis package [33]. Briefly, the paired end reads were denoised, dereplicated, and filtered for chimeras and joined together (DADA2) [34] and Scikit-learn [35] was used to taxonomically classify the resulting paired reads against the SILVA release 132 reference database [36–38] for 16S (prokaryote) analysis filtered at 99% identity. Reads not matching a reference sequence were removed from analysis (0.001%). OTUs were assigned based on a database hit of 99% or greater sequence identity and taxonomy was assigned against the database. Core diversity analysis was used to perform α-diversity and rarefaction and β-diversity and rarefaction functions. All resulting Fastq files can be accessed in the National Center for Biotechnology information (NCBI) under GenBank database BioProject Accession No. PRJNA862496.

### RNA-Seq analysis

Intestinal samples for RNA-Seq analysis were taken from a subset of fish at the end of the 56-day study period and placed in RNA Later (Invitrogen, Thermo Fisher Scientific, Waltham, MA, USA). Total RNA from tilapia anterior intestinal samples (1 cm posterior to the stomach) were extracted using standard methods with TRI Reagent (Molecular Research Centre, Inc., Cincinnati, OH, USA) and RNA integrity was verified with a BioAnalyzer 2100 (Agilent). The samples were then submitted to the NCSU Genomic Sciences Laboratory (Raleigh, NC, USA) for library preparation and RNA-Seq analysis using the Illumina HiSeq 2500 platform. For each treatment, a barcoded amplicon library was constructed and the pooled sample was run on a single Illumina lane (125 bp, single end reads). Due to low RNA quality in some individual samples, the pooled samples used to create the libraries for each treatment group consisted of n = 4 for 25% ST, 14-day/25% GO, n = 2 for 25% ST, 14-day/38% GO, n = 4 for 48% ST, 21-day/25% GO, and n = 4 for 48% ST, 21-day/38% GO. Following sequencing, the Fastq output files were trimmed for barcode removal and quality control. Mapping of the resulting reads to the tilapia genome [39] and subsequent identification of differentially expressed genes (DEGs) between each treatment group and the control group (48% ST, 21-day/38% GO) was conducted using CLC Genomics Workbench and the NCSU Bioinformatics Consulting Service Core high-performance computing cluster. The DAVID Bioinformatics suite [40] was then used to perform Gene Ontology (GO) term enrichment and KEGG pathway analysis of the DEG lists for each treatment group. All resulting Fastq files can be accessed in the National Center for Biotechnology information (NCBI) under BioProject Accession No. PRJNA607980.

### Statistical analysis

Length, weight, feed consumption, feed conversion ratio, and survival were analyzed for treatment effects at each time point by one-way ANOVA followed by a Tukey’s *post-hoc* test (GraphPad Prism 6; San Diego, CA, USA) or R 4.0.1 [41]. Alpha diversity was represented using the Shannon Index (species richness and relative abundance) and Faith’s PD (phylogenetic diversity) and statistically analyzed by Kruskal-Wallis tests. Weighted and unweighted Unifrac distances [42] were used to compute the beta diversity which was visualized using principal coordinates analysis (PCoA) plots with Emperor [43]. Significance was assigned *a priori* at an α of 0.05.

## Results

### Growth, feed conversion, and survival

**Fig 2:**
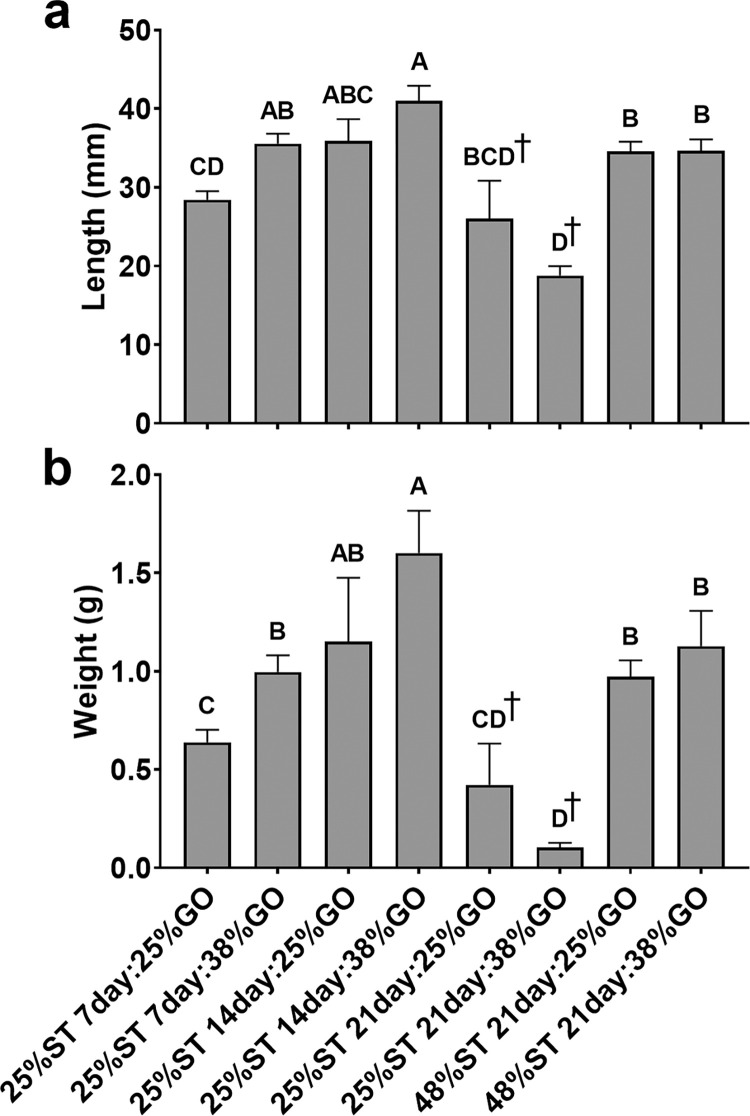
Final lengths (a) and weights (b) of Nile tilapia (*Oreochromis niloticus*) fry fed control and restricted protein diets after 56 days of culture. Data represented are means ± SEM. Labels indicate the number of days on the initial 25% or 48% starter (ST) protein diet followed by the growout (GO) diet containing 25% or 38% crude protein. Different letters indicate statistical significance in length and weight (one-way ANOVA; *P* < 0.05). ^†^Reflects mean values after 35 days of culture due to low survival in fish fed the 25% protein starter diet for 21 days. Growth parameters for different time points over the course of the culture period can be found in S3 Table.

Tilapia fry fed a starter diet containing 25% crude protein (CP) for 14 days and then provided a standard 38% CP growout diet thereafter had the highest final weight and lengths among all groups. Fish from this group had significantly higher lengths (40.89 ± 1.87 mm, mean ± SEM) and weights (1.60 ± 0.22 g, mean ± SEM) relative to fry fed a control 48% CP starter (ST) diet for 21 days followed by a 38% CP growout (GO) diet (34.66 ± 1.44 mm and 1.13 ± 0.18 g) (*P* < 0.005; Fig 2; S3 Table). Fry in the 25% ST, 7-day/38% GO diet group exhibited intermediate growth with a mean length of 35.56 ± 1.29 mm and weight of 1.00 ± 0.09 g, as did fry in the 25% ST, 14-day/25% GO group (35.92 ± 2.75 mm and 1.15 ± 0.33 g). Note that the final weights for fish in the 25% ST, 21-day group were taken after 35 days of culture rather than 56 days due to the high mortality rates but the trend toward smaller fish throughout the culture is evident (S3 Table).

**Fig 3:**
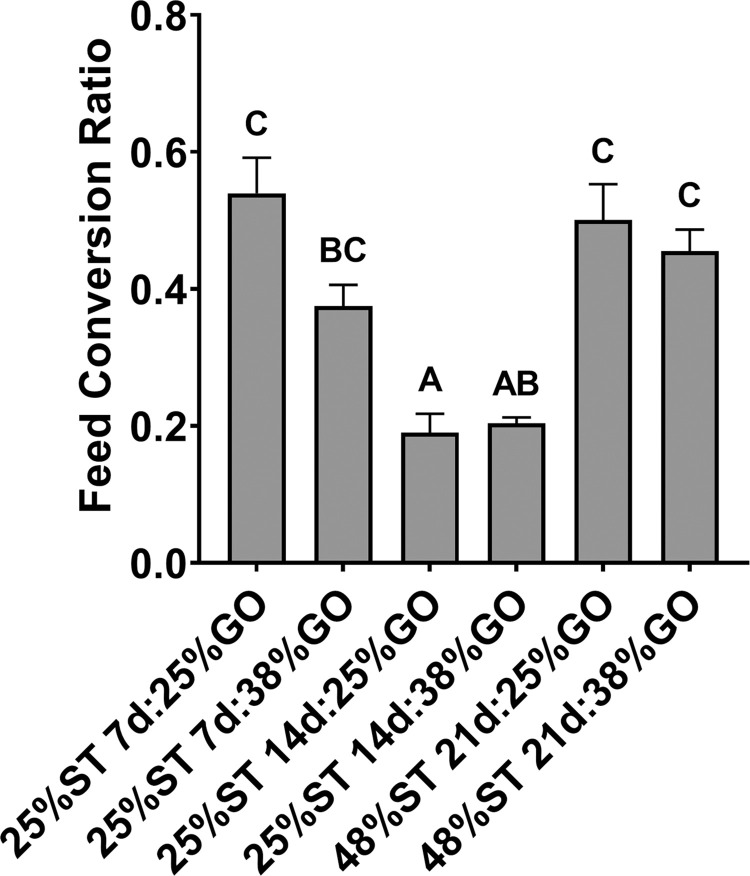
Feed conversion ratio of Nile tilapia (*Oreochromis niloticus*) initially fed restricted (25%) or control (48%) protein starter (ST) diets for 7, 14, or 21 days, followed by a protein restricted (25%) or control (38%) growout (GO) diet for the remainder of the 56-day study. Data are means ± SEM. Different letters indicate statistical significance (one-way ANOVA; *P* < 0.05).

Cumulative feed consumption was highest for fry fed the 48% ST and 25% or 38% GO diets, while animals fed the 25% ST diets and then the 25% GO diets had the lowest overall feed consumption (S3 Table). In addition to exhibiting the highest growth, both the 25% ST, 14-day/25% GO (0.19 ± 0.03, mean ± SEM) and 25% ST, 14-day/38% GO (0.20 ± 0.01, mean ± SEM) groups had significantly lower feed conversion ratios (FCRs) relative to all other groups after the 56-day trial (*P* < 0.005; Fig 3; S3 Table). Feed conversion for the 25% ST, 21-day groups (S3 Table) was calculated after 35 days of the trial versus after 56 days for the other treatments due to high mortality rates, see below.

**Fig 4:**
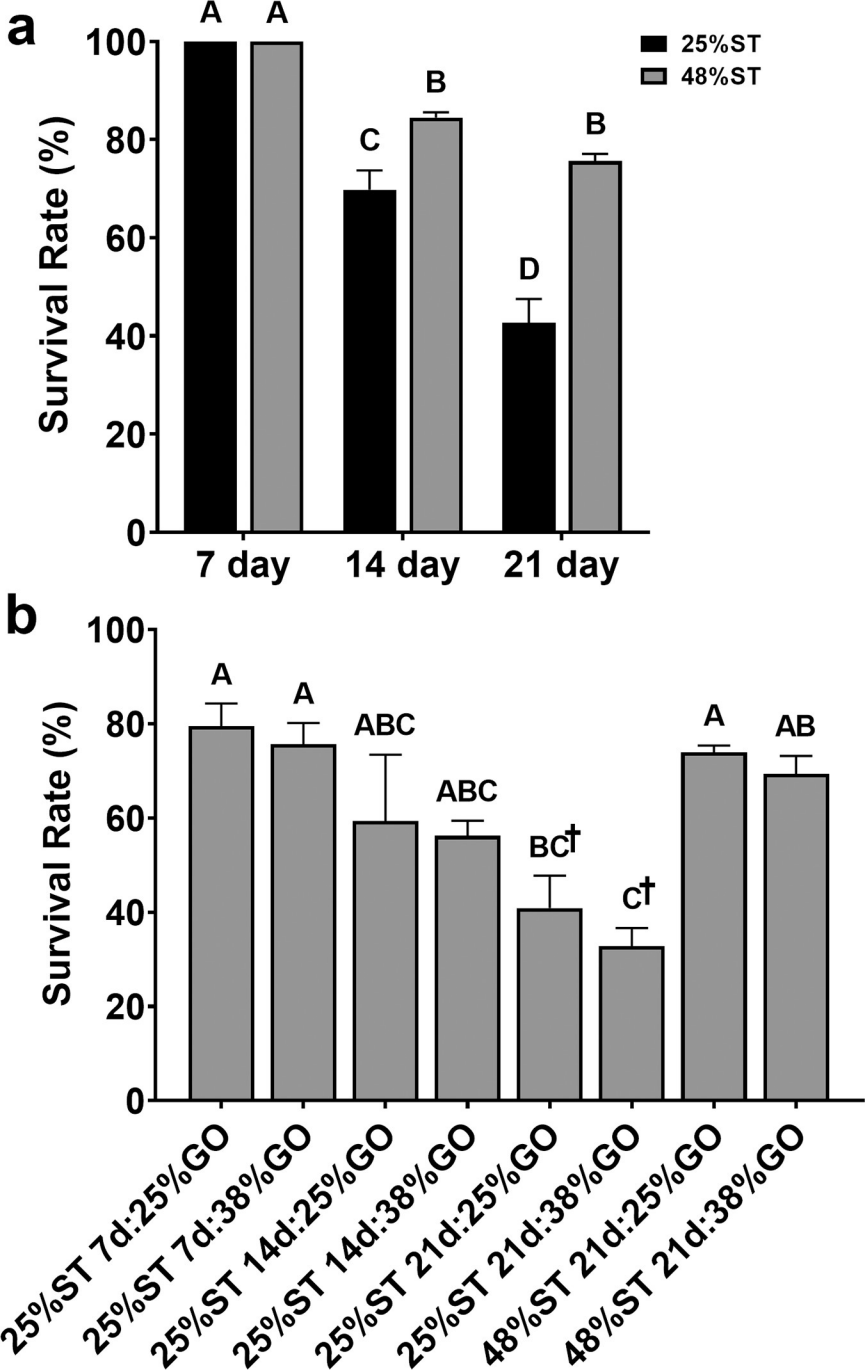
Survival rates (%) of Nile tilapia (*O*. *niloticus*) (a) initially fed restricted (25%) or control (48%) protein starter diets (ST) for 7, 14, or 21 days and (b) fed restricted (25%) or control (48%) protein starter diets for 7, 14, or 21 days, followed by a protein restricted (25%) or control (38%) growout (GO) diet for the remainder of the 56-day study. Data are means ± SEM. ^†^Reflects mean values after 35 days of culture due to low survival in fish fed the 25% protein starter diet for 21 days. Different letters indicate statistical significance (one-way ANOVA; *P* < 0.05).

No fry mortalities were observed for the first 10 days of the trial (Fig 4A), whilst the majority of mortalities within the study occurred between 10 and 21 days of the trial. For the 25% ST, 21-day treatment, regardless of growout diet given, the mortality rate was so high that those treatments were removed from the study at 35 days. Overall, we found that the survival rate decreased precipitously in the fish fed the 25% ST diet prior to switching to either of the growout diet, whereas the fry fed the 48% ST diet had a higher rate of survival through 21 days (*P* < 0.05; Fig 4A). By the end of the 56-day trial, there were no differences in survival of fish fed either the 25% ST, 7- or 14-day groups and the 48% ST groups regardless of the GO diet (Fig 4B).

### Microbiome analysis

**Fig 5:**
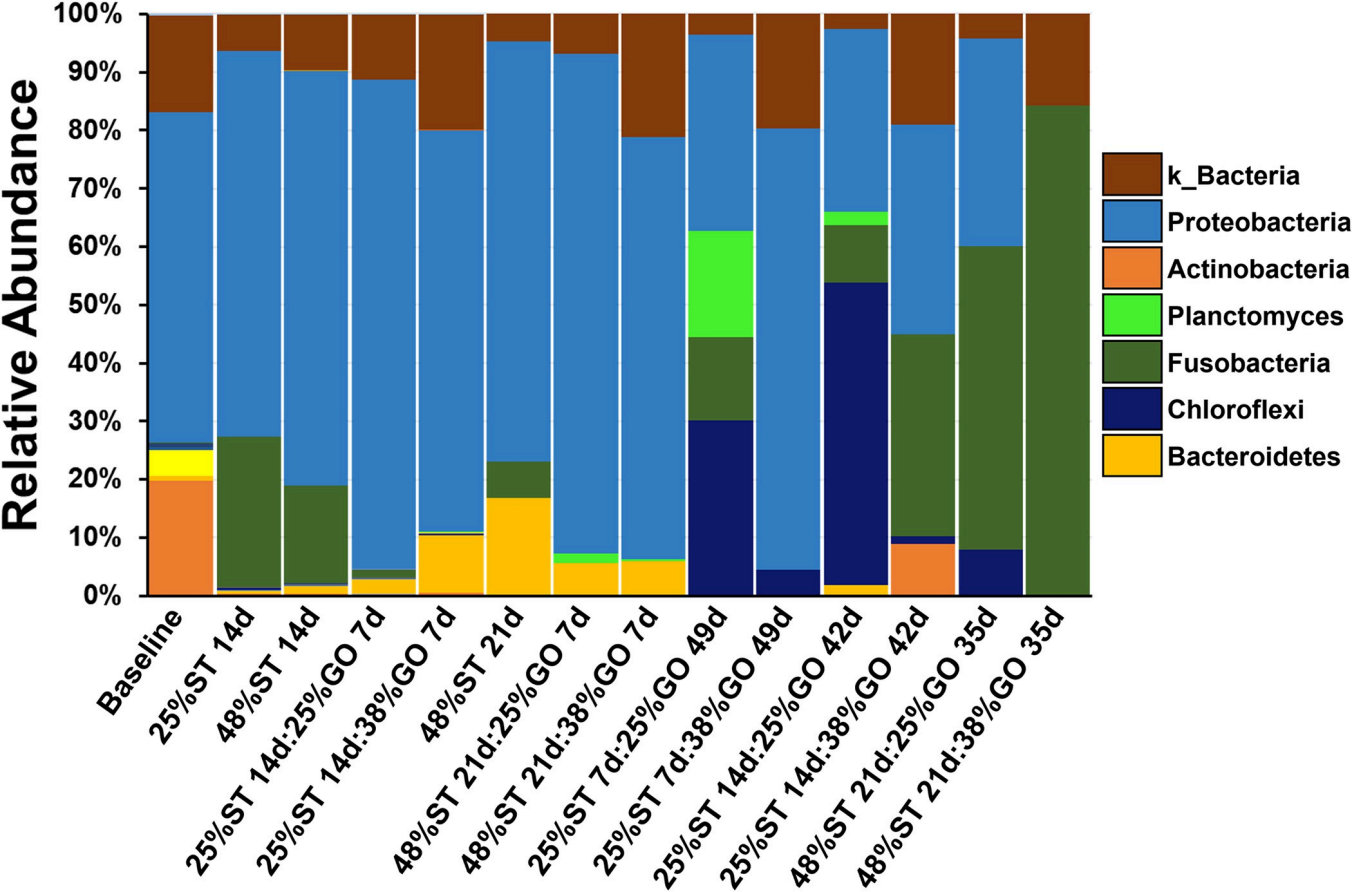
Relative abundance of the predominant operational taxonomic units (OTUs) at the phylum level from Nile tilapia (*Oreochromis niloticus*) posterior intestine at different stages of the growout period. k_Bacteria includes reads that could not be identified past Kingdom Bacteria. Labels indicate the number of days on the initial 25% or 48% protein starter (ST) diet followed by the number of days on the 25% or 38% protein growout (GO) diet. Baseline refers to samples from fish prior to first feeding.

We assessed how nutritional programming affects the gut microbial flora to determine whether such changes could benefit the growth and health of tilapia. After quality filtering and chimera removal, a total of 1,153,339 reads were obtained from sequencing the V3 to V5 regions of 16S rRNA prokaryotic gene (S4 and S5 Tables). There were 23 prokaryotic phyla, 44 classes, 112 orders, and 202 families associated with the tilapia used in this study of which 100% were Bacteria (no Archeae). Overall, the phyla Proteobacteria composed 70.0%, Fusobacteria 12.1%, Actinobacteria 3.1%, and Bacteroidetes 2.1% of the total reads. Reads that could not be classified beyond the Kingdom Bacteria composed 11.1%. Within the Proteobacteria, Gammaproteobacteria was the predominate class with genera *Aeromonas* comprising 10.1%, *Rheinheimera* 4.6%, *Shewanella* 1.3%, *Cellvibrio* 1.4%, *Pseudomonas* 1.7%, and *Enterovibrio* 14.0% of operational taxonomic units (OTUs). The relative abundance of identified phyla varied over time with the predominant phyla switching from Proteobacteria early on (≤ 28 days) to more Chloroflexi and Fusobacteria at the end of the study (Fig 5). There was less variation between treatments, however, the proliferation of Chloroflexi after 56 days appears to be more associated with groups in the restricted 25% ST diet while those fed the control 48% ST diet exhibited the highest increase in the proportion of Fusobacteria after 56 days (Fig 5).

**Fig 6:**
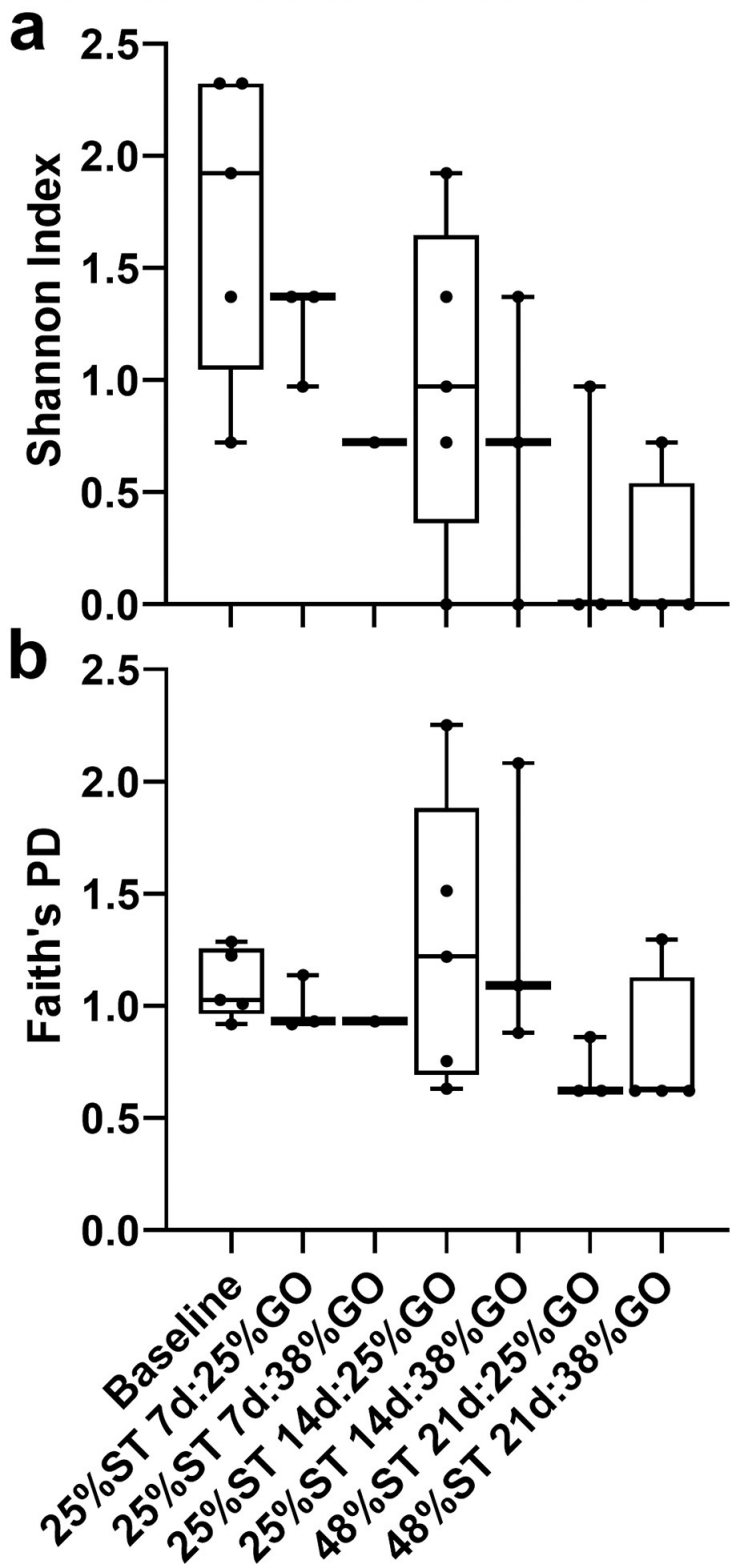
Alpha diversity of microbial flora in Nile tilapia (*Oreochromis niloticus*) posterior intestine prior to first feeding (baseline) or after 56 days of culture on diets with varying protein levels. Labels indicate the number of days on the initial 25% or 48% protein starter (ST) diets followed by the growout (GO) diet of 25% or 38% protein for the 56 day trial. (a) Shannon Index represents species richness and the relative abundance of those species within a sample. (b) Faith’s PD (phylogenetic diversity) represents the phylogenetic differences between species within a sample. No significant differences were observed for either analysis (Kruskal-Wallis; *P* = 0.059 and 0.207, respectively).

Alpha diversity measures indicate diversity within a community or sample. Operational taxonomic units are commonly used to estimate the species richness and abundance within each treatment based on the proportion of unique OTUs in each sample. We determined alpha diversity and rarefaction curves for all treatment groups. We examined two measures of alpha diversity for this study, the Shannon Index, which is a measure of species richness and the relative abundance of those species within a sample, and Faith’s phylogenetic diversity, which represents the phylogenetic differences between species within a sample (Fig 6). No significant differences in OTU richness were observed between fry fed the restricted or control protein diets with either the Shannon index (Kruskal-Wallis; *P* = 0.059; Fig 6A) or Faith’s phylogenetic diversity (Kruskal-Wallis; *P* = 0.207; Fig 6B). However, the Shannon Index depicts higher species richness, although not significant, in fry in the 25% ST group relative to those in the control 48% ST group (Fig 6A).

**Fig 7:**
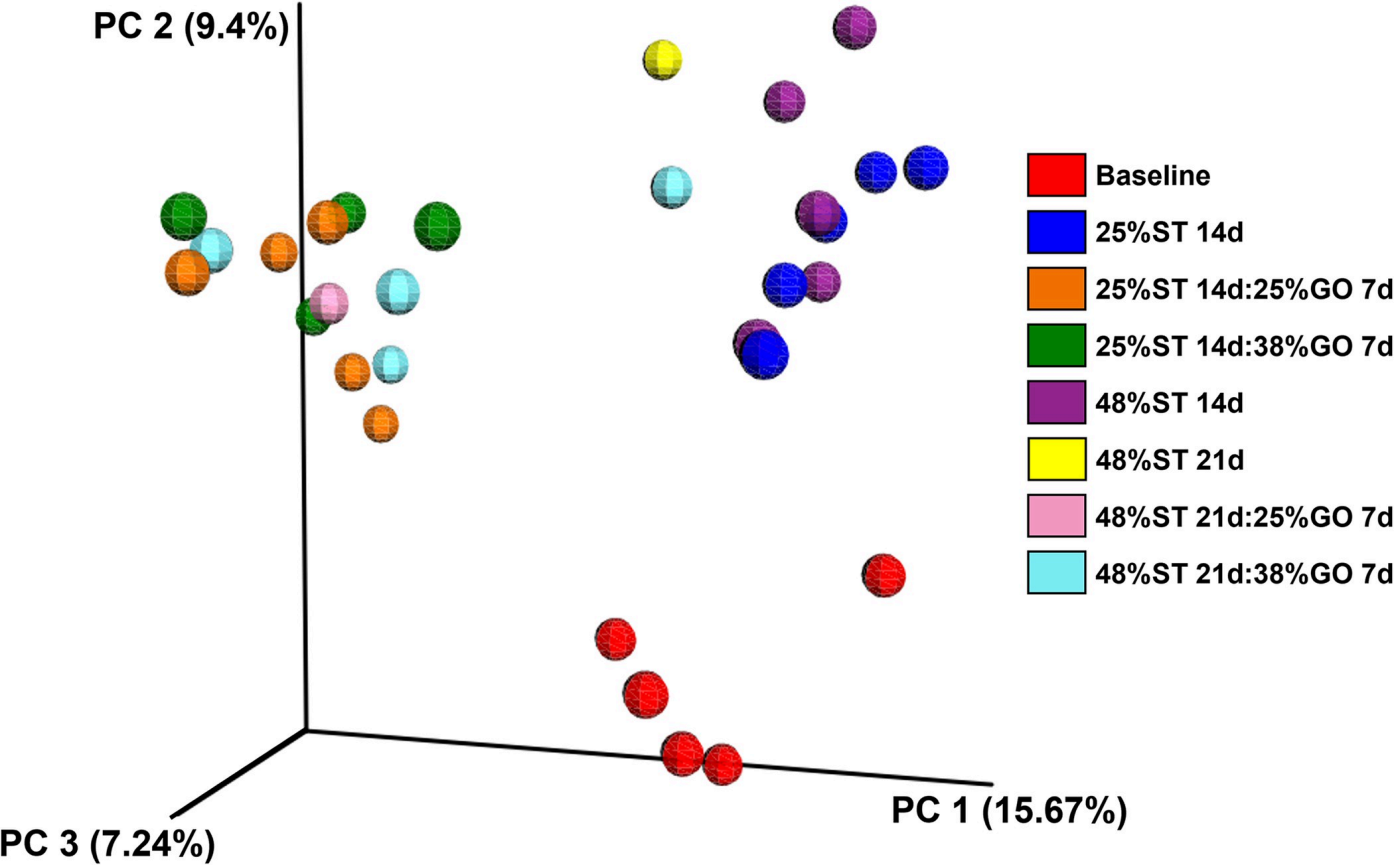
Principle coordinates analysis (PCoA) representing beta diversity of microbes identified in the Nile tilapia (*Oreochromis niloticus*) posterior intestine prior to first feeding (baseline) and after 14, 21, or 28 days of culture. Labels indicate the number of days on the initial 25% or 48% protein starter (ST) diets followed by the number of days on the 25% or 38% protein growout (GO) diets. Diversity was computed using unweighted Unifrac distances.

Beta diversity is a measure of the microbial diversity between treatments Fig 7. In our analysis, principle coordinate 1 explained 15.67% of the variability between samples, principle coordinate 2 explained 9.4% of the variability, and principle coordinate 3 explained 7.24% of the variability after 14, 21, and 28 days of culture (Fig 7). Variation in diversity of microbial communities was observed between the baseline fish, those fish on the starter diets, and those fish switched to the growout diets, regardless of protein content (Fig 7).

### RNA-Seq analysis

We investigated changes in the intestinal transcriptome between fry fed the control protein diet (48% ST, 21-day/38% GO) and those fed a 25% ST, 14-day/25% GO restricted diet, a 25% ST, 14-day/38% GO diet, or a 48% ST, 21-day/25% GO diet. The RNA-seq data were run against human, mouse, and zebrafish databases to identify gene names and perform gene ontology and Kyoto Encyclopedia of Genes and Genomes (KEGG) analyses but only the results from the human comparison will be reported here as it provided the greatest number of identities. In the 25% ST, 14-day/25% GO restricted group, there were a total of 1047 differentially expressed genes (DEGs) that could be identified by name, 316 of which had increased expression relative to the control diet and 749 that had decreased expression. In the 25% ST, 14-day/38% GO group, there were 38 DEGs identified by name with 24 having increased expression and 14 having decreased expression relative to the control diet. Lastly in the 48% ST, 21-day/25% GO group, there were 39 DEGs identified by name with increased expression compared to the control diet and 34 with decreased expression. All gene ontology terms and related genes can be found in S6 Table.

**Fig 8:**
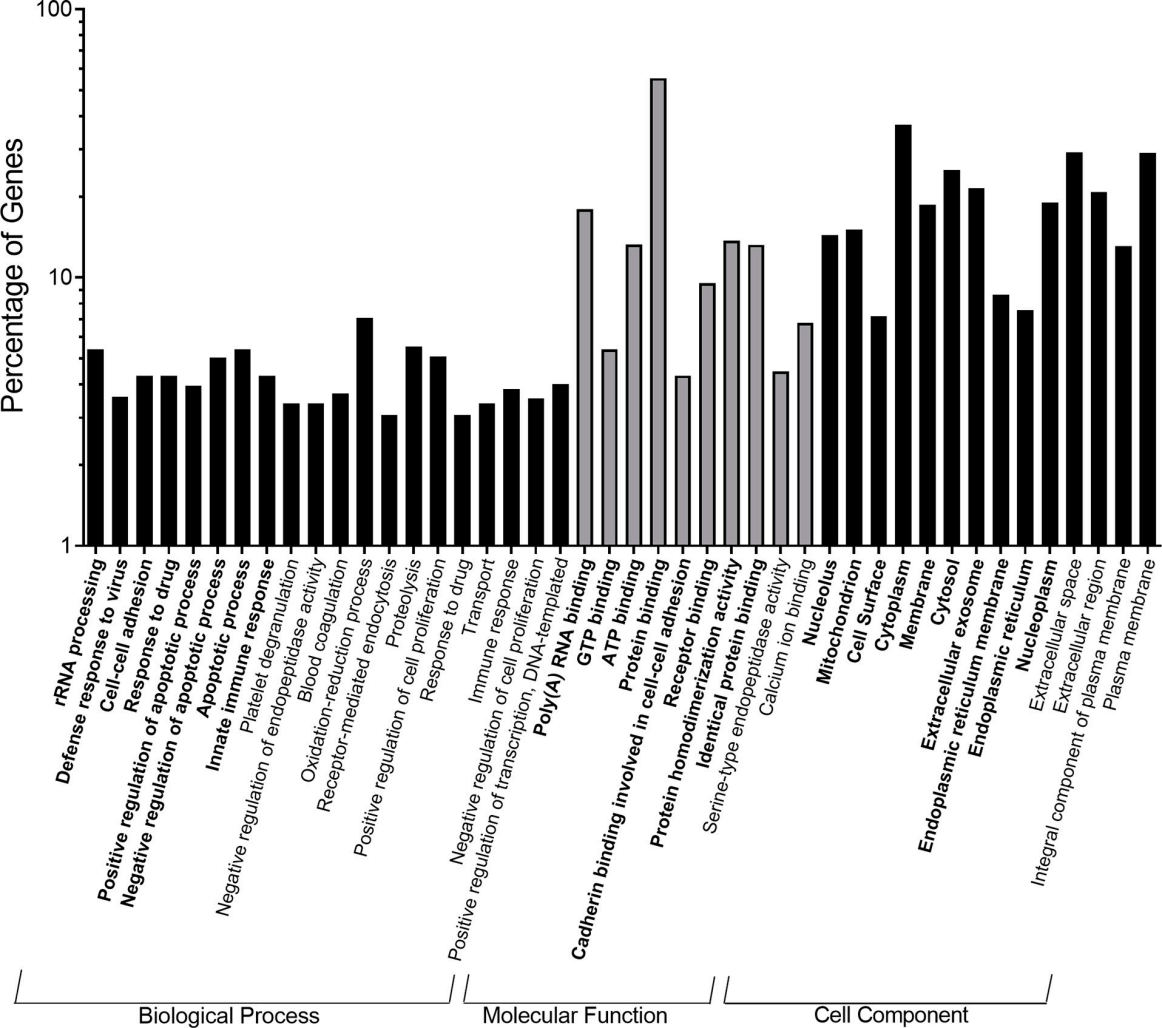
Top enriched gene ontology annotations of highly significant differentially expressed genes from the anterior intestine of Nile tilapia fry (*Oreochromis niloticus*) fed the restricted 25% crude protein starter diet followed by the 25% crude protein growout diet relative to the control diet (48% crude protein starter to 38% crude protein growout). The gene ontology terms are divided into the three major functional categories of biological process, molecular function, and cell component. Bold gene ontology terms were up-regulated relative to the control diet, gene ontology terms not bold and italicized were down-regulated relative to the control diet.

When upregulated genes were analyzed, there were 158 gene ontology terms enriched in the 25% ST 14 day/25% GO restricted group compared to controls (48% ST, 21-day/38% GO) with 68 falling under Biological Process (BP), 60 under Molecular Function (MF), and 30 under Cellular Component (CC) (Fig 8). In general, the Biological Process category composed the majority of DEGs for all comparisons and will be the primary focus of discussion. Many of the terms being enriched in the restricted group involved ribosomal RNA, transport RNA, and ribosome processing and assembly as well as protein folding and transport (Fig 8). Interestingly, we also identified numerous gene ontology terms involving immune cell activation, viral response, and cell adhesion that were enriched in the restricted group. The importance of these categories were confirmed with the KEGG analysis as the majority of the 11 pathways identified using upregulated genes involved ribosome and tRNA biogenesis as well as disease. Analysis of downregulated genes in the 25% ST, 14-day/25% GO group revealed the enrichment of 305 gene ontology terms (200 BP, 65 MF, and 40 CC) and 22 KEGG pathways. The most interesting terms and pathways fell under the broad categories of general metabolism, amino acid metabolism and biosynthesis, blood coagulation, and immune system complement cascades.

**Fig 9:**
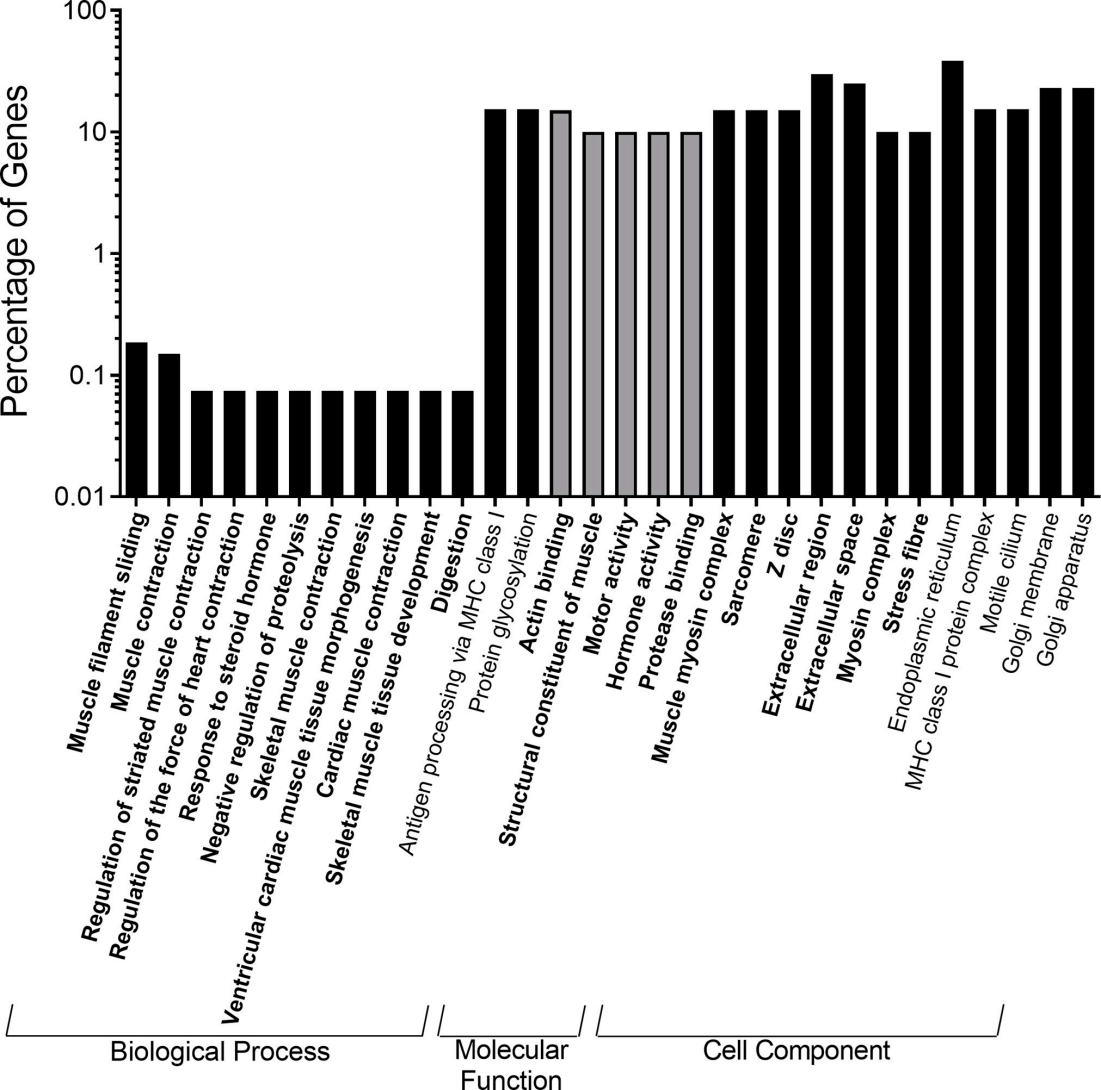
Top enriched gene ontology annotations of highly significant differentially expressed genes from the anterior intestine of Nile tilapia fry (*Oreochromis niloticus*) fed the restricted 25% crude protein starter diet followed by the 38% crude protein growout diet relative to the control diet (48% crude protein starter to 38% crude protein growout). The gene ontology terms are divided into the three major functional categories of biological process, molecular function, and cell component. Bold gene ontology terms were up-regulated relative to the control diet; gene ontology terms not bold and italicized were down-regulated relative to the control diet.

**Fig 10:**
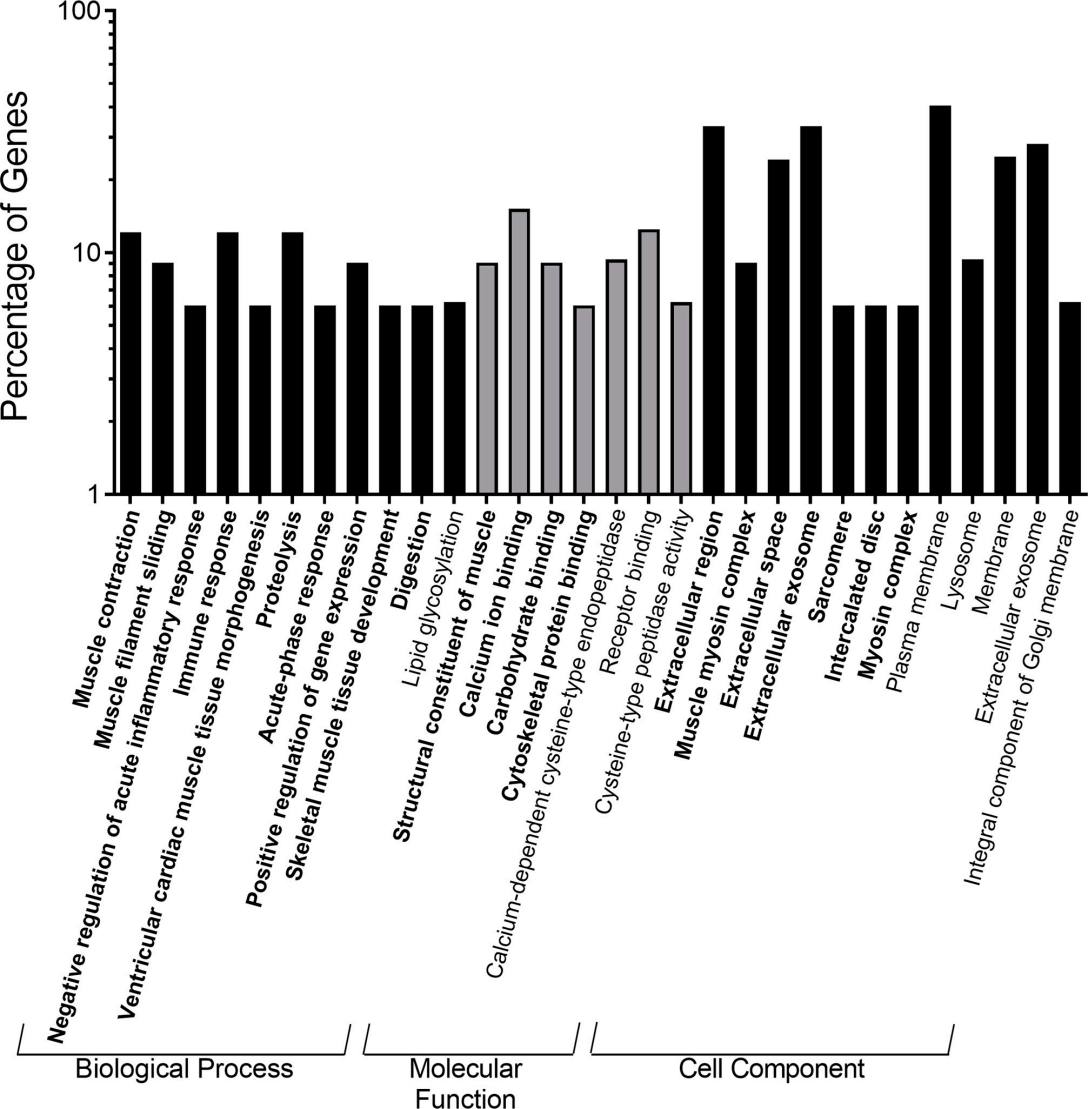
Top enriched gene ontology annotations of highly significant differentially expressed genes from the anterior intestine of Nile tilapia fry (*Oreochromis niloticus*) fed the 48% crude protein starter diet followed by the 25% crude protein growout diet relative to the control diet (48% crude protein starter to 38% crude protein growout). The gene ontology terms are divided into the three major functional categories of biological process, molecular function, and cell component. Bold gene ontology terms were up-regulated relative to the control diet; gene ontology terms not bold and italicized were down-regulated relative to the control diet.

In the 25% ST, 14-day/38% GO group, upregulated genes showed enrichment of 23 gene ontology terms (11 BP, 5 MF, and 7 CC) and 3 KEGG pathways. The majority of the BP terms and KEGG pathways were related to muscle function although the gene ontology terms negative regulation of proteolysis, response to steroid hormone, and digestion were also enriched (Fig 9). Downregulated genes in this group only enriched 7 gene ontology terms (2 BP and 5 CC) and there was no enrichment of KEGG pathways. The 2 BP terms identified were antigen processing and presentation of peptide and protein glycosylation. In the 48% ST, 21-day/25% GO group, upregulated genes showed enrichment of 21 gene ontology terms (10 BP, 4 MF, and 7 CC) but no KEGG pathways. Similar to the previous group, many of the BP terms that were enriched involved muscle contraction and development with immune response, proteolysis, and digestion also showing up in the analysis (Fig 10). Downregulated genes for the 48% ST, 21-day/25% GO group showed enrichment of 9 gene ontology terms (1 BP, 3 MF, and 5 CC) but again no KEGG pathways. The sole BP term enriched in this group was lipid glycosylation.

## Discussion

Nutritional programming has the potential to enhance nutrient utilization and feed efficiency in aquaculture. As protein reflects the costliest component of formulated feeds, reductions in its content could greatly enhance production efficiency for farmed tilapia. We examined the effects of restricting dietary protein for 7-, 14-, or 21-days post-yolk sac absorption in Nile tilapia and determined that providing a 25% crude protein (CP) starter (ST) diet for 7 or 14 days and then switching to a control 38% CP growout (GO) diet did not negatively impact growth or survival relative to fry fed a 48% CP diet initially for the 56-day trial (Figs 2 and 4). In fact, fry fed the lower protein diet for 14 days were significantly larger than the control fish after 56 days of culture (Fig 2) suggesting that the initial conditioning period allowed for better conversion of feed (Fig 3; S3 Table) and improved skeletal and somatic growth in these fish. Restricting protein for 21 days, however, led to both diminished growth and a significant reduction in survival rate (Figs 2 and 4), indicating that protein restriction should not be extended beyond 14 days post-yolk sac absorption. Interestingly, fry fed the 25% CP ST diet for 14 days followed by the 25% CP GO diet had similar final lengths and weights as fish in the 48% ST, 21-day groups (Fig 2) and significantly better feed conversion than fish in the 25% ST, 7-day groups and in the 48% ST, 21-day groups (Fig 3). Previous studies show tilapia may grow similarly on lower CP diets (25–27%) relative to higher protein diets [44, 45], albeit the effect may be diminished if tilapia are provided lower CP diets at fry stages (0.5 g) later than that tested here [44]. The results suggest that nutritional programming in tilapia is likely possible if protein restriction commences immediately following post-yolk sac absorption and is limited to under 14 days. The ability to lower the protein content in diets early has the potential to improve growth and enhance the efficiency of producing tilapia fry in tank culture. Even the use of lower protein growout diets have negligible impacts on growth following low protein nutritional programming. However, as the present study terminated at 56 days, we do not know whether the trend observed in growth with restriction of protein beginning at post yolk-sac absorption will continue until the fish reach marketable size.

The establishment of beneficial gut flora to increase nutrient absorption is an emerging research focus in human biology and aquaculture [46] and could augment existing practices of sustainable feeding and reduction in environmental footprint. Studies in Nile tilapia have shown that increases in intestinal microbial diversity are correlated with greater feed conversion [31]. In rainbow trout (*O*. *mykiss*), studies have shown diet-induced changes in the intestinal microbiome. Geurden and colleagues [16] observed differences in the intestinal fungi profiles in trout larvae fed a high glucose, low protein diet relative to those fed a high protein, glucose-free diet and these differences were sustained in the juvenile stage. Another study evaluated the effects of increasing proportions of dietary plant protein in juvenile trout and showed a significant change in the relative abundance of the different phyla as well as an overall decrease in alpha diversity with increasing plant protein levels [47]. In the present study, there were no major differences in alpha or beta diversity between fish initially fed the 25% CP diet and those fed the 48% CP diet and although the relative abundance of the identified phyla changed over time (shifting from Proteobacteria early on to more Chloroflexi and Fusobacteria), no diet-specific patterns emerged when comparing differences in protein content (Figs 5–7). While seemingly not the most interesting result, this indicates that in general, limiting dietary protein will not negatively impact the gut microbial flora in tilapia. Similarly, zebrafish (*Danio rerio*) fed dietary soybean meal in early developmental stages and then a fishmeal diet showed greater weight gain than fish fed fishmeal throughout but did not see any significant changes in the gut microbiome between treatment groups [48]. There were, however, differences in microbial community diversity when shifting the fry from the starter diets to the growout diets, regardless of the protein content given (Fig 7). This could be due to differences in the ingredient composition of the starter versus growout diets. The main differences between the formulations of the starter and growout diets were that the starter diets included bloodmeal and wheat gluten, while the growout diets did not. Diets containing blood meal or corn gluten meal have been found to have negative effects on alpha diversity in the distal intestine of the yellow kingfish (*Seriola lalandi*) [49]. However, plant-based protein sources in the diets of the olive flounder (*Paralichthys olivaceous*) had no effect on the microbial richness of the gut microbiota [50]. It is not currently known whether these contributed to the differences in microbial community diversity we observed here.

We did identify two potential organisms of concern in fry fed the 25% CP diet early on in the study as well as in the baseline samples (samples collected prior to the start of the feeding trials): *Mycobacterium sp*. and *Flavobacterium columnare* (see S4 and S5 Tables for reference). These both have the potential to be highly pathogenic in fish and tend to proliferate in infected culture systems. Mycobacteriosis is common in intensive aquaculture systems and can lead to weight loss and ulcers as well as cause zoonotic infections [51, 52]. *F*. *columnare* causes columnaris disease in freshwater fish which presents with skin and fin erosion and gill necrosis and often leads to death [53]. Neither species was identified in later samples once the fish had been switched to the growout diets, though. Interestingly, the baseline samples and the 25% ST, 14-day/38% GO samples were the only two groups in which Actinobacteria composed a discernible portion of the observed OTUs. This phylum is of great medical importance due to their antibacterial and antifungal properties [54, 55] but whether their appearance in our samples is related to the presence of pathogenic organisms or to the higher growth rate in 25% ST, 14-day/38% GO fish is unknown.

The final aspect of this study was to compare the intestinal transcriptomes between fish fed the control 48% ST, 21-day/38% GO diet and those that received restricted protein diets. In total, the 25% ST, 14-day/38% GO group showed enrichment of 30 gene ontology terms and 3 Kyoto Encyclopedia of Genes and Genomes (KEGG) pathways relative to the control group. The 48% ST, 21-day/25% GO group also showed enrichment of 30 gene ontology terms but there was no KEGG pathway enrichment in this group. This is in stark contrast to the fish that received the 25% ST, 14-day/25% GO restricted diet throughout the study which had a total of 463 enriched gene ontology terms and 33 enriched KEGG pathways relative to the control diet. This suggests that in general, long-term protein restriction has broader physiological effects and may be more detrimental to intestinal function than short-term protein restriction. In mammals, the small intestine is one of the most biologically active tissues and the splanchnic tissues retain 20–50% of dietary essential amino acids [56]. Similarly, the digestive tract of rainbow trout (*Oncorhynchus mykiss*) accounts for 39% of total protein synthesis ahead of both muscle (22%) and liver (14%) [57]. If the tilapia intestine is similarly active, this would explain the drastic changes in gene expression observed with the long-term reductions in dietary protein.

In the 48% ST, 21-day/25% GO group, the primary gene ontology terms being enriched were proteolysis, muscle contraction, immune response, and digestion (Fig 10). The upregulation of genes associated with proteolysis could indicate a restructuring of the intestine to provide amino acids for the production of more essential proteins. One possibility is the synthesis of proteins involved in muscle contraction as we see an upregulation of genes such as actin and myosin. This could act to maintain normal intestinal function and motility despite the reduction in protein availability with the 25% GO diet. In the 25% ST, 14-day/38% GO group, muscle contraction and immune responses were also enriched, along with cell signaling and negative regulation of proteolysis (Fig 9). The latter may indicate a compensatory mechanism wherein protein synthesis is enhanced due to sudden increases in dietary amino acids, perhaps programmed early on from the initial protein restriction.

The 25% ST, 14-day/25% GO restricted group had 1047 named DEGs, 463 enriched gene ontology terms, and 33 enriched KEGG pathways relative to the standard protein control diet (48% ST, 21-day/38% GO) indicating a drastic change in the intestinal physiology of this group of fish (Fig 8). Gene ontology terms enriched by the upregulated genes under the biological process category include rRNA processing and maturation, tRNA synthesis and migration, ribosome biogenesis and assembly, and protein folding and transport, which are all related to protein production, while notable molecular functions include poly(A) RNA binding, ATP binding, and protein binding. In weaned piglets, a low-protein diet led to impaired translation initiation and an overall reduction in the ability to synthesize proteins [58]. Given this, the upregulation of genes involved in protein synthesis and binding observed in our fish may serve to compensate for the decrease in dietary protein availability. By contrast, the downregulated genes enriched biological process gene ontology terms involved with amino acid catabolism and proteolysis and molecular functions related to protein binding, protein homodimerization, and endopeptidase activity. Again, this is to be expected since a lack of protein and thus amino acid availability would necessitate conservation of proteins and amino acids. An interesting result was the number of enriched gene ontology terms and KEGG pathways that were related to cell adhesion, response to viruses, and immune function. The upregulation of genes involved in cell-to-cell recognition and adhesion may indicate a reduced ability to synthesize tight junction proteins which could increase epithelial permeability to unwanted materials as has been observed in weanling rats [59]. Dietary protein restriction also impairs T-cell mediated immune responses and reduce the ability to kill bacteria in leopard frog tadpoles (*Lithobates sphenocephalus*) [60] and decreases immunity to gastrointestinal parasites in rodents and small ruminants [61–65]. Further, fasting significantly altered expression of genes related to innate immune function in channel catfish, *Ictalurus punctatus* [66] and a notable term being enriched in our study was the response to starvation. This indicates that a reduction in dietary protein may simulate the fasted state, thus contributing further to the altered immune function. As mentioned above, we identified *Mycobacterium sp*. and *Flavobacterium columnare* in 25% CP restricted fish at the earliest time points in the study and this could also be a factor leading to the upregulation of genes related to general immune function. Gene expression of components of the inflammatory response has been found to be upregulated in adult zebrafish due to chronic *M*. *marinum* infections [67] and of immune-related genes in channel catfish following *F*. *columnare* challenge [68]. Thus, while feeding fry a low protein diet for the full 56 days had no negative impacts on growth and would further reduce feed-associated production costs, there appear to be trade-offs such that intestinal function and immune responses may be significantly impaired which could affect growth and health later in the growout stage.

The mechanisms that may underlie nutritional programming remain poorly understood [5]. Although our study did not identify a specific mechanism for improved growth, it may have been due to several factors such as morphological alteration of the intestine, increased nutrient absorption at a post-transcriptional level, alterations to regulation of digestive hormones, or to changes in metabolism or energy partitioning [69]. It is also possible that epigenetic changes of the DNA may influence expression of genes involved in protein utilization and synthesis [5, 70]. Epigenetic modifications made early could affect gene expression throughout the life of the fish and may even be heritable. Further research examining the mechanisms of nutritional programming associated with enhanced growth, including potential epigenetic impacts, require further study.

Overall, restricting dietary protein (25% ST) for the first 14 days post-yolk sac absorption in Nile tilapia led to a significant increase in lengths and weights and better feed efficiency after 56 days of culture relative to fry fed normal protein diets (48% ST, 21-day/38% GO). This suggests a type of compensatory growth in which the initial lack of protein may lead to better uptake and utilization of this nutrient later on. Further, the protein restricted diet showed no negative impacts on the gut microbial flora and the effects on the intestinal transcriptome were limited. Thus, our results indicate that tilapia are likely responsive to nutritional programming and that it may be possible to increase production efficiency by reducing the levels of protein in starter feeds for fry shortly after post yolk-sac absorption.

## Supporting information

S1 Table Nutrient composition of standard (48% or 38% crude protein) and restricted (25% crude protein) starter and growout diets used for nutritional programming of Nile tilapia (*Oreochromis niloticus*). ME, metabolizable energy. DE, digestible energy.
(PDF)

S2 Table Primer sequences used for the microbiome analysis. (PDF)

S3 Table Lengths (mm), weights (g), and cumulative feed consumption (g) of Nile tilapia (*Oreochromis niloticus*) over the course of the 56-day culture period. Data are means ± SEM. Column headings indicate the protein content and number of days on the starter diet followed by the protein content of the growout (GO) diet. Letters indicate significant differences (*P* < 0.05).
(PDF)

S4 Table 16S rRNA amplicon sequence data feature table output from the QIIME2 workflow indicating all observations for each barcoded sample submitted for sequencing. Column headings indicate the protein content and number of days on the starter diet followed by the protein content of the growout (GO) diet.
(XLSX)

S5 Table 16S rRNA amplicon sequence data taxonomy classifications from the QIIME2 workflow using the SILVA 132 database. Column 1 lists all feature IDs referenced in the feature table output, column 2 lists the closest aligned OTU (operational taxonomic unit) to the sequences, and column 3 lists the confidence of the alignment.
(XLSX)

S6 Table Full list of genes identified in the RNA-Seq analysis. Genes are divided by diet group and classified by gene ontology term.
(XLSX)
