# Supplementary material for: Nutritional programming in Nile tilapia (Oreochromis niloticus): Effect of low dietary protein on growth and the intestinal microbiome and transcriptome
Source: PLoS One. 2023 Oct 4;18(10):e0292431. doi: 10.1371/journal.pone.0292431 (PMC10550151; doi:10.1371/journal.pone.0292431)
Supplement: S1 Table — ME, metabolizable energy. DE, digestible energy. (PDF) [file pone.0292431.s001.pdf]

S1 Table. Nutrient composition of standard (48% or 38% crude protein) and restricted (25% crude protein) starter and growout diets used for nutritional programming of Nile tilapia (*Oreochromis niloticus*). ME, metabolizable energy. DE, digestible energy.

	25% Starter	48% Starter	25% Growout	38% Growout
Dietary Ingredients	-- % of Diet --			
**Menhaden Fishmeal**	10.08	24.67	9.85	8.47
**Poultry products meal**	0	19.74	0	14.05
**Hydrolyzed Feather Meal**	6.72	7.24	6.24	7.7
**Wheat flour middlings**	33.59	9.87	49.24	23.42
**Wheat grain, hard red**	25.19	14.8	14.77	15.06
**Dehulled Soybeans, 50% CP**	1.68	9.87	9.85	25.1
**Spray Dried Bloodmeal**	5.04	4.93	0	0
**Wheat Gluten**	1.01	1.64	0	0
**Menhaden Fish Oil**	3.02	2.96	3.94	4.35
**Soybean Oil**	7.73	0	3.28	0
**Mono Calcium Phosphate**	2.15	0	1.31	0.5
**Vitamin and Mineral Premix**[1]	2.52	2.47	1.23	1.25
**Vitamin C**	0.44	0.43	0.04	0.04
**Choline Chloride 60%**	0.24	0.13	0.13	0.07
**L-Lysine, 95%**	0.6	1.11	0.1	0
**DL-Methionine, 99%**	0	0.15	0.02	0
**Total**	100	100	100	100
Calculated Analysis				
**Crude Protein, %**	25.00%	48.00%	25.00%	38.00%
**Crude Fat, %**	12.13%	8.58%	9.59%	8.00%
**Total Carbohydrates, %**	36.72%	15.88%	36.59%	23.80%
**Crude Fiber, %**	3.64%	2.04%	4.78%	3.96%
**Ash, %**	4.47%	6.38%	5.17%	6.15%
**ME (Trout)**	2883 kcal/kg	2790 kcal/kg	2639 kcal/kg	2599 kcal/kg
**ME (Catfish)**	2584 kcal/kg	1986 kcal/kg	2352 kcal/kg	2350 kcal/kg
**DE (Trout)**	3177 kcal/kg	3254 kcal/kg	2934 kcal/kg	2973 kcal/kg
**DE (Catfish)**	3200 kcal/kg	3244 kcal/kg	2970 kcal/kg	2974 kcal/kg
**Total Digestibility**	73.96%	73.49%	76.44%	76.56%
**HUFA & PUFA, %**	0.59%	0.59%	0.79%	0.83%
**Cholesterol, %**	0.05%	0.12%	0.05%	0.10%
**Total Calcium, %**	0.93%	1.92%	0.82%	0.66%
**Total Phosphorous, %**	1.24%	1.56%	1.20%	1.17%
**Arginine, %**	1.18%	2.41%	1.41%	1.60%
**Lysine, %**	1.96%	3.80%	1.45%	2.19%
**Methionine, %**	0.45%	1.08%	0.44%	0.66%
**Cysteine, %**	0.35%	0.74%	0.37%	0.63%
**Taurine, %**	0.00%	0.00%	0.00%	0.05%
**Threonine, %**	0.75%	1.53%	0.75%	1.21%
**Tryptophan, %**	0.30%	0.50%	0.31%	0.48%
**Valine, %**	1.09%	2.00%	0.96%	1.08%
**Choline Chloride**	3000 mg/kg	3000 mg/kg	2500 mg/kg	2500 mg/kg

[1] Premix supplied by Integral Fish Foods, Inc. (Albany, IN, USA) provides a proprietary blend of vitamins, minerals, and other additives that meet or exceed the recommendations of the Nutrient Requirements of Fish and Shrimp (National Research Council, 2011) and Mjoun et al. (2010).
