# Supplementary material for: Nutritional programming in Nile tilapia (Oreochromis niloticus): Effect of low dietary protein on growth and the intestinal microbiome and transcriptome
Source: PLoS One. 2023 Oct 4;18(10):e0292431. doi: 10.1371/journal.pone.0292431 (PMC10550151; doi:10.1371/journal.pone.0292431)
Supplement: S2 Table — (PDF) [file pone.0292431.s002.pdf]

**S2 Table. Primer sequences used for the microbiome analysis.**

| ***Primer*** | ***Primer Sequence*** | ***Primer Usage*** |
|---|---|---|
| 357FMod | 5'-TCGTCGGCAGCGTCAGATGTGTATAAGAGACAGCCTACGGGAGGCAGCAG-3' | 16S Amplicon Primer |
| 926RMod | 5'-GTCTCGTGGGCTCGGAGATGTGTATAAGAGACAGCCGTCAATTCMTTTRAGT-3' | 16S Amplicon Primer |
| N701 | 5'-CAAGCAGAAGACGGCATACGAGATTCGCCTTAGTCTCGTGGGCTCGG-3' | Index 1 Primer |
| N702 | 5'-CAAGCAGAAGACGGCATACGAGATCTAGTACGGTCTCGTGGGCTCGG-3' | Index 1 Primer |
| N703 | 5'-CAAGCAGAAGACGGCATACGAGATTTCTGCCTGTCTCGTGGGCTCGG-3' | Index 1 Primer |
| N704 | 5'-CAAGCAGAAGACGGCATACGAGATGCTCAGGAGTCTCGTGGGCTCGG-3' | Index 1 Primer |
| N705 | 5'-CAAGCAGAAGACGGCATACGAGATAGGAGTCCGTCTCGTGGGCTCGG-3' | Index 1 Primer |
| N706 | 5'-CAAGCAGAAGACGGCATACGAGATCATGCCTAGTCTCGTGGGCTCGG-3' | Index 1 Primer |
| N707 | 5'-CAAGCAGAAGACGGCATACGAGATGTAGAGAGGTCTCGTGGGCTCGG-3' | Index 1 Primer |
| N708 | 5'-CAAGCAGAAGACGGCATACGAGATCCTCTCTGGTCTCGTGGGCTCGG-3' | Index 1 Primer |
| N709 | 5'-CAAGCAGAAGACGGCATACGAGATAGCGTAGCGTCTCGTGGGCTCGG-3' | Index 1 Primer |
| N710 | 5'-CAAGCAGAAGACGGCATACGAGATCAGCCTCGGTCTCGTGGGCTCGG-3' | Index 1 Primer |
| N501 | 5'-AATGATACGGCGACCACCGAGATCTACACTAGATCGCTCGTCGGCAGCGTC-3' | Index 2 Primer |
| N502 | 5'-AATGATACGGCGACCACCGAGATCTACACCTCTCTATTCGTCGGCAGCGTC-3' | Index 2 Primer |
| N503 | 5'-AATGATACGGCGACCACCGAGATCTACACTATCCTCTTCGTCGGCAGCGTC-3' | Index 2 Primer |
| N504 | 5'-AATGATACGGCGACCACCGAGATCTACACAGAGTAGATCGTCGGCAGCGTC-3' | Index 2 Primer |
| N505 | 5'-AATGATACGGCGACCACCGAGATCTACACGTAAGGAGTCGTCGGCAGCGTC-3' | Index 2 Primer |
| N506 | 5'-AATGATACGGCGACCACCGAGATCTACACACTGCATATCGTCGGCAGCGTC-3' | Index 2 Primer |
| N507 | 5'-AATGATACGGCGACCACCGAGATCTACACAAGGAGTATCGTCGGCAGCGTC-3' | Index 2 Primer |
| N508 | 5'-AATGATACGGCGACCACCGAGATCTACACCTAAGCCTTCGTCGGCAGCGTC-3' | Index 2 Primer |
