# Supplementary material for: Nutritional programming in Nile tilapia (Oreochromis niloticus): Effect of low dietary protein on growth and the intestinal microbiome and transcriptome
Source: PLoS One. 2023 Oct 4;18(10):e0292431. doi: 10.1371/journal.pone.0292431 (PMC10550151; doi:10.1371/journal.pone.0292431)
Supplement: S3 Table — Data are means ± SEM. Column headings indicate the protein content and number of days on the starter diet followed by the protein content of the growout (GO) diet. Letters indicate significant differences (P < 0.05). (PDF) [file pone.0292431.s003.pdf]

S3 Table. Lengths (mm), weights (g), and cumulative feed consumption (g) of Nile tilapia (*Oreochromis niloticus*) over the course of the 56-day culture period. Data are means ± SEM. Column headings indicate the protein content and number of days on the starter diet followed by the protein content of the growout (GO) diet. Letters indicate significant differences ($P < 0.05$).

	**25%, 7 day: 25% GO**	**25%, 7 day: 38% GO**	**25%, 14 day: 25% GO**	**25%, 14 day: 38% GO**	**25%, 21 day: 25% GO**	**25%, 21 day: 38% GO**	**48%, 21 day: 25% GO**	**48%, 21 day: 38% GO**
Length (mm)								
**0 days**	9.47 ± 0.14	9.47 ± 0.14	9.47 ± 0.14	9.47 ± 0.14	9.47 ± 0.14	9.47 ± 0.14	9.47 ± 0.14	9.47 ± 0.14
**7 days**	10.62 ± 0.18[b]	10.62 ± 0.18[b]	---	---	---	---	12.80 ± 0.44[a]	12.80 ± 0.44[a]
**14 days**	11.59 ± 0.41[b]	15.01 ± 0.78[a]	14.04 ± 0.79[a]	14.04 ± 0.79[a]	---	---	13.87 ± 0.48[a]	13.87 ± 0.48[a]
**21 days**	14.27 ± 1.30[c]	18.64 ± 1.45[abc]	15.95 ± 0.49[bc]	18.59 ± 0.63[ab]	15.62 ± 0.62[c]	15.62 ± 0.62[c]	18.82 ± 0.84[a]	18.82 ± 0.84[a]
**28 days**	14.55 ± 0.98[b]	21.66 ± 1.17[a]	18.23 ± 0.83[ab]	22.29 ± 1.94[a]	16.04 ± 0.62[ab]	18.06 ± 1.74[ab]	21.02 ± 1.26[a]	19.10 ± 0.76[ab]
**56 days**	28.42 ± 1.09[cd]	35.56 ± 1.29[ab]	35.92 ± 2.75[abc]	40.89 ± 1.87[a]	26.00 ± 4.49 [bcd †]	18.77 ± 1.31 [d †]	34.56 ± 1.25[b]	34.66 ± 1.44[b]
Weight (g)								
**0 days**	0.012 ± 0.001	0.012 ± 0.001	0.012 ± 0.001	0.012 ± 0.001	0.012 ± 0.001	0.012 ± 0.001	0.012 ± 0.001	0.012 ± 0.001
**7 days**	0.016 ± 0.011[b]	0.016 ± 0.011[b]	---	---	---	---	0.029 ± 0.003[a]	0.029 ± 0.003[a]
**14 days**	0.023 ± 0.006[b]	0.061 ± 0.012[a]	0.038 ± 0.008[ab]	0.038 ± 0.008[ab]	---	---	0.040 ± 0.005[ab]	0.040 ± 0.005[ab]
**21 days**	0.073 ± 0.007[ab]	0.096 ± 0.007[ab]	0.064 ± 0.008[b]	0.102 ± 0.012[ab]	0.061 ± 0.010[b]	0.061 ± 0.010[b]	0.123 ± 0.018[a]	0.123 ± 0.018[a]
**28 days**	0.106 ± 0.007[ab]	0.157 ± 0.009[a]	0.130 ± 0.006[a]	0.162 ± 0.014[a]	0.061 ± 0.008[b]	0.109 ± 0.036[ab]	0.197 ± 0.041[a]	0.120 ± 0.018[ab]
**56 days**	0.638 ± 0.064[c]	0.996 ± 0.086[b]	1.151 ± 0.325[ab]	1.601 ± 0.215[a]	0.422 ± 0.195 [cd †]	0.104 ± 0.024 [d †]	0.973 ± 0.082[b]	1.128 ± 0.179[b]
Cumulative Feed Consumption (g)								
**7 days**	3.80 ± 0.08[b]	3.80 ± 0.08[b]	---	---	---	---	4.20 ± 0.06[a]	4.20 ± 0.06[a]
**14 days**	5.09 ± 1.03[b]	6.85 ± 0.04[a]	6.75 ± 0.09[a]	6.75 ± 0.09[a]	---	---	7.72 ± 0.23[a]	7.72 ± 0.23[a]
**21 days**	11.32 ± 1.08[b]	13.28 ± 0.12[ab]	10.47 ± 3.02[b]	14.15 ± 0.92[ab]	13.09 ± 0.27[ab]	13.09 ± 0.27[ab]	16.48 ± 0.45[a]	16.48 ± 0.45[a]
**28 days**	23.10 ± 1.13[ab]	25.59 ± 0.04[a]	17.83 ± 3.31[b]	24.16 ± 0.92[ab]	24.34[‡]	23.79[‡]	29.10 ± 1.11[a]	29.44 ± 0.38[a]
**35 days**	37.19 ± 1.04[b]	39.80 ± 0.51[ab]	26.86 ± 2.93[c]	35.84 ± 0.98[b]	33.96[‡]	32.01[‡]	44.62 ± 1.33[a]	45.11 ± 0.60[a]
**56 days**	72.32 ± 1.75[b]	82.59 ± 3.09[ab]	51.58 ± 3.68[c]	71.81 ± 0.91[b]	---	---	91.31 ± 1.44[a]	90.06 ± 1.91[a]
Feed Conversion Ratio (FCR)								
**7 days**	4.00 ± 2.54	4.00 ± 2.54	---	---	---	---	0.96 ± 0.23	0.96 ± 0.23
**14 days**	4.24 ± 2.04	0.43 ± 0.09	6.09 ± 5.15	6.09 ± 5.15	---	---	1.02 ± 0.24	1.02 ± 0.24
**21 days**	0.60 ± 0.03	0.48 ± 0.01	0.76 ± 0.12	0.53 ± 0.09	65.68 ± 30.25*	65.68 ± 30.25*	0.44 ± 0.10	0.44 ± 0.10
**28 days**	0.64 ± 0.04[ab]	0.43 ± 0.01[a]	0.35 ± 0.04[a]	0.42 ± 0.03[a]	20.34*	9.14*	0.42 ± 0.05[a]	0.88 ± 0.16[b]
**56 days**	0.54 ± 0.05[c]	0.37 ± 0.03[bc]	0.19 ± 0.03[a]	0.20 ± 0.01[ab]	38.91*	27.09*	0.50 ± 0.05[c]	0.46 ± 0.03[c]

[†] 25% ST, 21-day/25% GO and 25% ST, 21-day/38% GO length and weight measurements are given after 35 days of the trial due to low survivability of the fry in these treatments.

[‡] Not included in statistical analysis due to the low survival of the 25% CP starter 21-day treatment groups.

* Approximate FCR due to low survival of the 25% CP starter 21-day treatment groups. Not included in statistical analysis
